# On measuring agreement with numerically bounded linguistic probability schemes: A re-analysis of data from Wintle, Fraser, Wills, Nicholson, and Fidler (2019)

**DOI:** 10.1371/journal.pone.0248424

**Published:** 2021-03-18

**Authors:** David R. Mandel, Daniel Irwin

**Affiliations:** 1 Intelligence, Influence and Collaboration Section, Toronto Research Centre, Defence Research and Development Canada, Toronto, Ontario, Canada; 2 Department of National Defence, Toronto, Ontario, Canada; University of Defence in Belgrade, SERBIA

## Abstract

Across a wide range of domains, experts make probabilistic judgments under conditions of uncertainty to support decision-making. These judgments are often conveyed using linguistic expressions (e.g., *x* is *likely*). Seeking to foster shared understanding of these expressions between senders and receivers, the US intelligence community implemented a communication standard that prescribes a set of probability terms and assigns each term an equivalent numerical probability range. In an earlier PLOS ONE article, [1] tested whether access to the standard improves shared understanding and also explored the efficacy of various enhanced presentation formats. Notably, they found that embedding numeric equivalents in text (e.g., *x* is *likely* [55–80%]) substantially outperformed the status-quo approach in terms of the percentage overlap between participants’ interpretations of linguistic probabilities (defined in terms of the numeric range equivalents they provided for each term) and the numeric ranges in the standard. These results have important prescriptive implications, yet Wintle *et al*.’s percentage overlap measure of agreement may be viewed as unfairly punitive because it penalizes individuals for being *more* precise than the stipulated guidelines even when the individuals’ interpretations fall perfectly within the stipulated ranges. Arguably, subjects’ within-range precision is a positive attribute and should not be penalized in scoring interpretive agreement. Accordingly, in the present article, we reanalyzed Wintle *et al*.’s data using an alternative measure of percentage overlap that does not penalize in-range precision. Using the alternative measure, we find that percentage overlap is substantially elevated across conditions. More importantly, however, the effects of presentation format and probability level are highly consistent with the original study. By removing the ambiguity caused by Wintle et al.’s unduly punitive measure of agreement, these findings buttress Wintle et al.’s original claim that the methods currently used by intelligence organizations are ineffective at coordinating the meaning of uncertainty expressions between intelligence producers and intelligence consumers. Future studies examining agreement between senders and receivers are also encouraged to reflect carefully on the most appropriate measures of agreement to employ in their experiments and to explicate the bases for their methodological choices.

## Introduction

Across a variety of domains, experts make probabilistic judgments under conditions of uncertainty to support decision-making (e.g., [2–7]). For instance, clinicians estimate the likelihood of certain treatment outcomes, meteorologists predict the weather, and intelligence analysts forecast geopolitical developments. Depending on the context, uncertainty may stem from the sense that critical information is missing or inconsistent with other pieces of information, doubts about the accuracy or completeness of one’s causal model, awareness of the possibility of benign misinformation or malign deception, and even aversion to the risk of being proven wrong. In order to fulfill the decision-support function, expert assessors must not only be able to estimate their uncertainty to arrive at sound judgments, they must also effectively communicate those probabilistic judgments to their respective audiences. Without effective communication, even an accurate and timely judgment could potentially misinform the end-user.

Often, experts (and the professional or organizational bodies they may represent) communicate probabilistic judgments using linguistic expressions such as *likely* or *very likely* for probability levels that convey “more likely than not”; conversely, experts use terms such as *unlikely* or *very unlikely* to convey probability levels “less likely than not.” Political pundits, for example, are prone to using such language in their forecasts without specifying what the linguistic probabilities mean to them [8]. Although the free use of such language is still tolerated in some intelligence communities [9], others have implemented communication schemes aimed at fostering agreement; namely, shared meaning between senders and receivers.

In the US, for instance, the Office of the Director of National Intelligence promulgated a communication standard under [10] that prescribes a set of probability terms and assigns each term an equivalent numerical probability range (see Table 1). In practice, intelligence consumers are expected to reference a translation table to ensure that their interpretations of linguistic probabilities agree with the prescribed meanings. The North Atlantic Treaty Organization (NATO) and the UK intelligence community follow the same basic approach, although the precise set of terms and numerical ranges assigned differ [11–13]. Numerically bounded linguistic probability (NBLP) schemes are used in many other domains as well. For instance, the Intergovernmental Panel on Climate Change (IPCC) uses this approach to communicate probabilistic assessments about global climate change [14] and the European Commission recommends a similar system for communicating the risk of drug side effects [15]. NBLP schemes have also been proposed for use in extreme event attribution reports ([16]; for a critique, see [17]).

**Table 1:** Linguistic probability expressions and numeric range equivalents in [10].

Almost no chance	Remote	1–5%
Very unlikely	Highly improbable	5–20%
Unlikely	Improbable (improbably)	20–45%
Roughly even chance	Roughly even odds	45–55%
Likely	Probable (probably)	55–80%
Very likely	Highly probable	80–95%
Almost certain(ly)	Nearly certain	95–99%

Note: ICD 203 provides two sets of linguistic probability expressions that are treated synonymously. In [1], participants were only shown terms from the first column and their numeric equivalents.

With the burgeoning adoption of NBLP schemes by a variety of governmental and other organizations, a growing body of research has examined the extent to which NBLP schemes improve shared understanding or agreement. Such research has explored the efficacy of alternative presentation formats for linguistic probabilities. Notably, [5,18,19] have examined the NBLP guideline used by the IPCC. This work has consistently shown that providing end-users access to a translation table only marginally improves agreement between the end-user and the guideline. They have further demonstrated that including numeric range equivalents in text beside linguistic probabilities (e.g., *x* is *likely* [>66%]) yields better agreement than using the standard alone, but even this hybrid method of presenting linguistic probabilities with numeric probability ranges leaves considerable room for improvement. For instance, measuring percentage overlap from 0% (if the participant’s interpretation fell completely outside the stipulated range) to 100% (if the participant’s interpretation was fully contained), [19] found that mean percentage overlap was 18.5% among participants provided the translation table, compared to 33.6% among participants provided in-text numeric translations.

In an important extension of [5,18,19] work, [1] examined the efficacy of the US intelligence community guideline shown in Table 1. Aside from noteworthy differences between the two NBLP schemes (e.g., some numeric ranges overlap in the IPCC standard but are mutually exclusive in ICD 203), Wintle *et al*. compared the effect of a greater number of presentation formats (described in the next section) on the percentage of overlap between participants’ interpretations of linguistic probabilities (defined in terms of the numeric range equivalents they provided for each term) and the numeric ranges in ICD 203. Compared to a control condition, they found that percentage overlap (i.e., a measure of agreement) significantly improved only in the hybrid condition in which numeric range equivalents were shown next to the linguistic probability phrase in text.

Wintle *et al*.’s (2019) [1] findings are important because they strongly generalize the findings of [5,18,19] studies from the domain of climate science to that of intelligence analysis. Given that the intelligence community, like many areas of government, is often slow to implement pan-organizational reform and to leverage relevant behavioral science [20,21], it is important to demonstrate that the limitations of NBLP schemes do not only exist in other expert communities, such as climate science. Nevertheless, the value of [1] research might be minimized because the measure of percentage overlap (PO) that they used is arguably unfairly punitive from a prescriptive perspective. The effect of unwarranted punitiveness in the measure of agreement may be used to challenge the validity of [1] findings and their policy relevance. Accordingly, we sought to address this issue and thereby resolve ambiguity regarding the earlier findings by introducing what we shall argue is a fairer measure of agreement based on proportion of range overlap.

Wintle *et al*.’s (2019) [1] calculated PO between participants’ interpreted ranges and the ICD 203-stipulated ranges as follows:

$$PO=\frac{min(U_{e},U_{s})-max(L_{e},L_{s})}{max(U_{e},U_{s})-min(L_{e},L_{s})}\times 100,$$ (1)

where *L*_*e*_, *B*_*e*_ (used subsequently), and *U*_*e*_ refer to the participant’s elicited lower-bound, best, and upper-bound numeric probability equivalents for a linguistic probability term used in a particular statement, respectively; and where the subscripts *e* and *s* refer to “elicited” and “stipulated”, respectively. This agreement measure penalizes individuals who provide compliant “in-range” interpretations that are *more precise* than the stipulated numeric ranges, or what [22] described as *nested intervals*. For instance, given that the stipulated range for *likely* is 55–80%, a participant interpreting *likely* as 60–75% would have a PO equal to 60% instead of 100%, as one might expect for such a nested interval. That is, in this example, agreement would be calculated as follows:

$$PO=\frac{min(75,80)-max(60,55)}{max(75,80)-min(60,55)}\times 100=60.$$

However, the precision exacted by such nested intervals can (and perhaps even ought to) be seen as a positive communication attribute insofar as precision tends to be valued by individuals facing judgment and decision-making tasks and will, in fact, tradeoff with accuracy to influence overall assessments of communication quality [23,24]. Penalizing precision where the receiver’s interpreted range is a fully nested set of the stipulated range may also distort the intended use of the NBLP scheme under examination. That is, when an intelligence analyst selects a term such as *likely*, it is only meant to signify that the analyst’s credible interval falls *within* the stipulated range (55–80%), not necessarily that it *equals* the full range.

Therefore, the PO measure of agreement used in [1] leaves a gap in our understanding of the degree of agreement achieved by the alternative presentations formats that these authors examined when a fairer measure of agreement that does not penalize nested intervals is employed. We address this gap by reanalyzing the data from Wintle *et al*. using an alternative PO measure of agreement that does not penalize participants for being more precise than the stipulated guidelines when their ranges fall within such guidelines. We achieve this result by scoring the percentage of the participant’s range that is covered by the stipulated range. In contrast to [1] symmetric measure of PO, our proposed measure is asymmetric in the sense that it takes the participant’s range as the reference point and scores how well the stipulated range accounts for the participant’s interpretation. Specifically, we define agreement as follows:

$$PO=\frac{1-[max(U_{e}-U_{s},0)+max(L_{e}-L_{s},0)]}{U_{e}-L_{e}}\times 100.$$ (2)

Given that our PO measure of agreement is less punitive, we hypothesized that we would observe a greater level of agreement across presentation methods. We further sought to test the hypothesis that the relative differences between presentation formats would remain unchanged. If this hypothesis were confirmed, it would serve to strengthen confidence in the conclusions drawn from [1] regarding the effectiveness of the alternative methods for communicating probability information.

## Materials and methods

Most of the relevant materials and methods are reported in full detail in [1]. However, for ease of reference, we summarize key aspects of their experiment and then detail the procedures we used for re-analyzing their data. Wintle *et al*. recruited 924 adult participants from a pool of 4,122 people who had expressed interest in a larger research project. The experiment was administered online via Qualtrics. Participants were randomly assigned to one of four presentation conditions: Table, Tooltip, Brackets, or Control. In the Table condition, participants could click on a link, opening a separate tab/window containing the ICD 203 NBLP scheme. In the Tooltip condition, participants could position their cursor over the probability term appearing in a statement, revealing the term’s numeric equivalent. In the Brackets condition, numeric range equivalents were embedded in text alongside verbal probability expressions (e.g., “likely [55–80%]”). The brackets condition is identical to what we earlier referred to as the hybrid format. In the Control condition, participants received no guidance on the stipulated numeric equivalents of each probability expression (i.e., they were not shown the ICD 203 guideline in any form).

Across presentation conditions, participants were shown eight statements presented in random order that were extracted from US intelligence reports. Each statement contained one of four probability terms (very unlikely, unlikely, likely, very likely) drawn from ICD 203 (e.g., “ISIS is ***unlikely*** to announce that it is ending its self-declared caliphate…”). Each probability term was presented twice, each time in a different statement. In our subsequent analyses, we average results over these two instantiations for each term, as the specific statements are not of theoretical interest. After reviewing a statement, participants were asked, “What do you think the authors mean by [probability term]?” Using a 101-point percentage-chance slider scale ranging from 0 (No chance) to 100 (Certain), participants gave their minimum, best, and maximum numerical probability equivalents.

### Analysis

Data and other supporting files associated with our re-analysis are available at https://osf.io/v9q82/.

We treated missing data differently than [1]. Where participants provided maximum estimates for the terms *unlikely* and/or *very unlikely* but were missing minimum estimates, Wintle *et al*. recoded the missing values as 0. This procedure was based on the assumption that these participants had intended to input 0 but did not realize that they had to move the slider from its default position (this procedure is not described in the original article, but is outlined in their code: https://osf.io/cp5rq/). Although this explanation is plausible, there is in fact no way to ascertain the extent to which this reason accounts for those missing cases. Accordingly, we adopted the more conservative procedure of treating all missing cases as truly missing.

Our data-handling procedures also differed from [1] in another respect. In cases where participants violated the “logical” constraint, *L*_*e*_ < *B*_*e*_ < *U*_*e*_, [1] rearranged their responses to conform to that inequality. Our use of scare quotes in the preceding sentence reflects the fact that the logical constraint referenced by [1] should instead be the relaxed expression, *L*_*e*_ ≤ *B*_*e*_ ≤ *U*_*e*_. That is, a participant giving a best estimate equal to one of the bounds would be unable to satisfy the strict inequality. More importantly, given that the bases of such incoherence are unknown, we do not believe this procedure is justified and [1] do not provide a justification for the intervention. Accordingly, we omitted 190 cases (2.6% of the sample) in which participants provided a minimum estimate that exceeded their maximum estimate. We omitted these cases because they cannot be meaningfully used to calculate the PO measure. However, best estimates that fell outside the participant’s lower and upper bounds (354 or 5.3% of valid cases) were left unchanged because this form of incoherence does not prohibit the calculation of any measure that was used in our re-analysis.

In contrast to [1], we calculated PO following Eq 2. Note that if spread (i.e., the width of the range) was equal to 0 (i.e., *L*_*e*_ = *U*_*e*_), which occurred in 95 cases, PO was equal to 100% if the value of the bounds was within the stipulated range and it was equal to 0% if the value of the bounds was outside the stipulated range.

To facilitate comparison with [1,5,17,18] also analyzed the proportion of participants’ best estimates falling within the stipulated ranges. To minimize the effect of outliers [25] and ensure consistency with earlier studies (e.g., [5]), we Winsorized best estimates to focus on the central 90 percent of the distribution within each of the 32 cells defined by the 4 (probability term) × 2 (intelligence statement) × 4 (presentation format) design.

## Results and discussion

### Missing data

There was a maximum of 7,392 responses to each of the three questions (i.e., minimum, best, and maximum estimates) in the experiment; namely, 924 participants each of whom were asked to assess eight statements. Overall, 13.26% (2,941/22,176) of responses were missing. Table 2 shows the percentage of missing responses by estimate, probability term, and presentation format. Each of the three types of estimates was independent of presentation format, all *p* >.2. In contrast, each of the three types of estimates was not independent of probability term. Chi-square test values (with *df* = 3 and *N* = 7,392) for minimum, best, and maximum estimates are 115.09, 12.42, and 14.02, respectively, all *p* < .01.

**Table 2:** Percentage of missing responses by estimate, probability term, and presentation format.

Factor/Level	Estimate		
Format	Minimum	Best	Maximum
Table	17.32	10.61	12.61
Tooltip	16.33	9.61	11.72
Brackets	17.10	11.69	12.99
Control	17.64	10.28	11.23
**Term**			
Very Unlikely	23.76	10.44	11.96
Unlikely	18.83	10.12	14.45
Likely	14.61	12.55	11.53
Very Likely	11.20	9.09	10.61

The pattern of missing responses for estimates of minimum and maximum values across probability term was comparable. In both cases, there was a greater percentage of missing data for the low probability terms *unlikely* and *very unlikely* than for the high probability terms *likely* and *very likely*. We note that although the result for minimum values is consistent with [1] assumption that missing data reflected a failure to adjust the slider in order to set it to zero, this would not explain why the same pattern of missing data was observed for the estimates of maximum value. The effect of probability term on missing responses for best estimates, however, showed a different pattern in which the term *likely* had a higher percentage of missing responses.

### Agreement as percentage overlap

**Fig 1:**
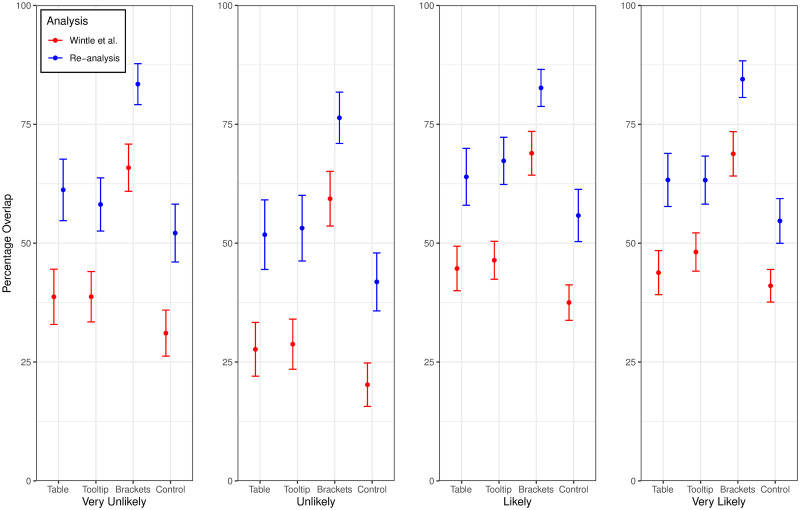
Mean percentage overlap (PO). Error bars show 95% confidence intervals around the means. Results are plotted as a function of presentation format (i.e., Table, Tooltip, Brackets, and Control conditions), probability term (i.e., Very Unlikely, Unlikely, Likely, and Very Likely), and analysis type ([1] in red and our re-analysis in blue).

We conducted a mixed (Presentation Format × Probability Term) analysis of variance (ANOVA) on our PO measure of agreement. The effect of probability term was significant, *F*(3, 472) = 25.07, *p* < .001, η_p_^2^ = .137. There was also a significant effect of presentation format, *F*(3, 474) = 41.30, *p* < .001, η_p_^2^ = .207. However, the interaction effect was not significant, *F*(9, 1422) = 1.08, *p* = .37, η_p_^2^ = .007. Tables 3 and 4 show mean PO as a function of presentation format and probability level, respectively, and Fig 1 plots PO as a function of these factors. As Table 3 shows, all pairwise comparisons between levels of presentation format were significant with the exception of the comparison between the Table and Tooltip conditions. As Table 4 shows, all pairwise comparisons among levels of probability term were significant with the exception of the comparison between the *likely* and *very likely*. Reproducing [1] findings, overlap was markedly worse for the phrase *unlikely*. Overall, as Fig 1 shows, the asymmetric PO measure used in our reanalysis was less punitive than the symmetric measure used by [1]. However, perhaps more importantly, it revealed a highly consistent pattern of results regarding the effects of the independent variables.

**Table 3:** Mean percentage overlap (PO) by presentation format.

		95% confidence interval	
Format	Mean	LB	UB
Table	60.10 ^a^	55.79	64.32
Tooltip	60.46 ^a^	56.14	64.79
Brackets	81.73 ^b^	77.89	85.58
Control	51.12 ^c^	46.90	55.33

Note: Superscripts in the “Mean” column denote a significant difference at the *p* = .05 probability level. LB and UB stand for lower and upper bounds, respectively.

**Table 4:** Mean percentage overlap (PO) by probability term.

		95% confidence interval	
Term	Mean	LB	UB
Very Unlikely	63.73 ^a^	60.95	66.50
Unlikely	55.79 ^b^	52.61	58.97
Likely	67.43 ^c^	64.91	69.94
Very Likely	66.43 ^c^	64.06	68.80

Note: Superscripts in the “Mean” column denote a significant difference at the *p* = .05 probability level. LB and UB stand for lower and upper bounds, respectively.

### Agreement as the percentage of in-range best estimates

**Fig 2:**
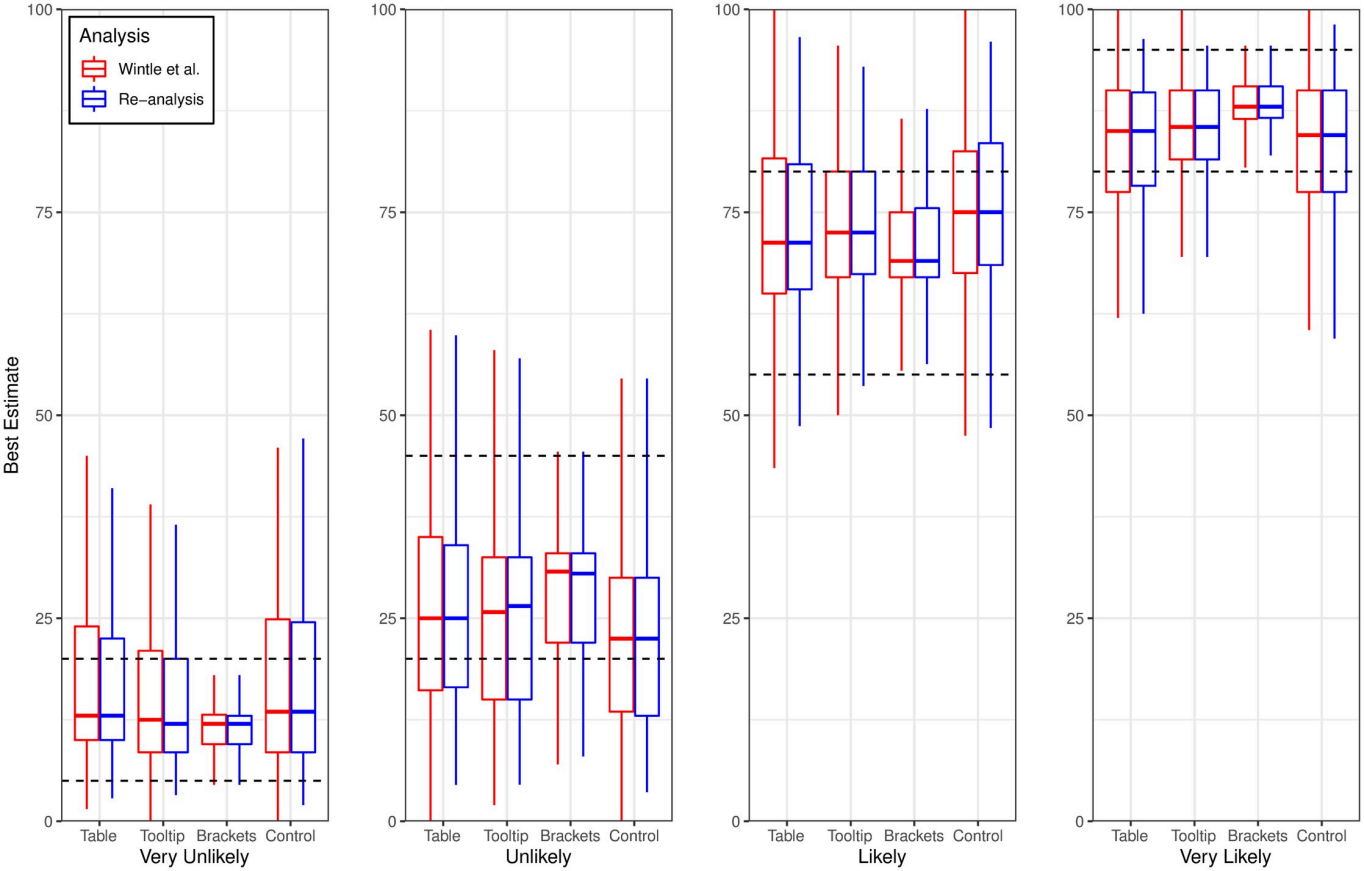
Box plots showing the median percentage of participant’s best estimates that agree with ICD 203 (the stipulated ranges are demarcated by the dashed lines). Results are plotted as a function of presentation format (i.e., Table, Tooltip, Brackets, and Control conditions), probability term (i.e., Very Unlikely, Unlikely, Likely, and Very Likely), and analysis type ([1] in red and our re-analysis in blue).

We conducted a mixed (Presentation Format × Probability Term) ANOVA on the percentage of best estimates that fell within the ranges specified in ICD 203. The effect of probability term on the percentage of best estimates that agreed with the ICD 203 standard was significant, *F*(3, 638) = 49.51, *p* < .001, η_p_^2^ = .189. There was also a significant effect of presentation format, *F*(3, 640) = 25.76, *p* < .001, η_p_^2^ = .108. However, the interaction effect was not significant, *F*(9, 1920) = 1.03, *p* = .41, η_p_^2^ = .005. Tables 5 and 6 show the mean percentage of best estimates that agreed with the ICD 203 standard as a function of presentation format and probability level, respectively, and Fig 2 plots the percentage of agreeing best estimates as a function of these factors. As Table 5 shows, the pairwise differences perfectly paralleled those obtained on the PO measure of agreement; namely, all conditions significantly differed except for the Table and Tooltip conditions. The pairwise differences between levels of probability term showed some discrepancies with those reported for the PO measure. Once again, agreement was poorest for the phrase *unlikely* (see Table 6). However, compliance was significantly better for the term *very likely* than for the remaining terms. Fig 2 shows how our results closely parallel those of [1]. This is to be expected given that our analyses differ mainly in terms of the data exclusion rules we applied and also the fact that we Winsorized the data.

**Table 5:** Mean percentage of best estimates that agree with the ICD 203 standard by presentation format.

		95% confidence interval	
Format	Mean	LB	UB
Table	66.50 ^a^	62.45	70.56
Tooltip	67.71 ^a^	63.70	71.73
Brackets	83.36 ^b^	79.53	87.18
Control	59.74 ^c^	55.81	63.67

Note: Superscripts in the “Mean” column denote a significant difference at the *p* = .05 probability level. LB and UB stand for lower and upper bounds, respectively.

**Table 6:** Mean percentage of best estimates that agree with the ICD 203 standard by probability term.

		95% confidence interval	
Format	Mean	LB	UB
Very Unlikely	73.56 ^a^	70.76	76.36
Unlikely	55.41 ^b^	52.14	58.67
Likely	70.88 ^a^	68.00	73.77
Very Likely	77.46 ^c^	74.86	80.05

Note: Superscripts in the “Mean” column denote a significant difference at the *p* = .05 probability level. LB and UB stand for lower and upper bounds, respectively.

## Conclusions

Breakdowns in the communication of uncertainty have played a significant role in major intelligence failures in recent history [26–29]. Wintle *et al*. (2019) [1] made an important contribution to the evaluation of the current standard used by the US intelligence community for communicating uncertainty in intelligence products. Their research showed that the NBLP scheme currently used did not promote a level of shared understanding one would expect for communications of great importance that could affect national security. Nevertheless, their method of scoring agreement could be judged to be overly punitive and their treatment of data followed procedures that rest on questionable assumptions. These features of the original experiment, therefore, may be interpreted as casting doubt on the validity of the findings.

To address this valid concern, we reanalyzed the data of [1] by using more conservative data exclusion rules and a less punitive PO measure of agreement. We did so not because we view Wintle *et al*.’s method as wrong, but rather because we believe it is fair to construe it as overly punitive from a prescriptive vantage point. We further believe that proponents of status-quo probability communication methods within the intelligence community might draw upon this characteristic and dismiss the validity of the research on that basis. Indeed, as noted earlier, the in-range precision that [1] PO measure scores as non-overlap might be regarded as a beneficial attribute given that receivers are often willing to trade accuracy for informativeness in the form of greater precision [23,24]. The PO measure that we used instead is not susceptible to the same critique. Although our asymmetric PO measure of agreement does not reward precision within the numeric probability bounds stipulated in ICD 203, it does not penalize it either.

Our reanalysis of agreement resulted in an elevated percentage overlap across conditions. This is to be expected given that our measure is less punitive than [1] measure. However, critically, the effect of presentation format and probability level was highly consistent with that reported in [1]. We found that the Table and Tooltip methods outperformed the control condition, and these methods were outperformed by the Brackets method. This ordering suggests that the more numeric representations are present at the time of interpretation, the more faithfully the sender’s interpretation is decoded. These results further suggest that the methods intelligence organizations currently use, which are closest to the Table condition in the present research, are unlikely to effectively sync the meaning of uncertainty expressions assigned by intelligence producers and inferred by intelligence consumers, such as policymakers who must rely on uncertain estimates to support critical decision-making about national security matters [11,30]. Even by our less punitive measurement standards, agreement based on our PO measure was approximately 60% in the Table condition, a mere 9% above the control condition, which would represent the intelligence community’s approach prior to the adoption of the ICD 203 standard. Similarly, if we compare the Table condition to the control group in terms of the agreement of participants’ best estimates, there is only a small increase from approximately 60% to 67%.

By comparison, the improvement in communication fidelity between the current intelligence community method and the enhanced method of including stipulated numeric probability ranges directly in assessments was far more substantial by both agreement metrics. This is consistent with the findings of [5,18,19]. Although these findings suggest that use of bracketed numeric ranges in estimates would improve communication fidelity, we urge caution in our recommending this strategy. One potential problem with the hybrid approach is that end-users might interpret numeric ranges as credible intervals on the substantive assessment given in an intelligence report. For example, imagine that an intelligence analyst states, “It is likely [55%-80%] that the Blanks will attack our country in the next week.” According to the hybrid method, the range given is meant to clarify what the term *likely* means, in general—namely, as outlined in ICD 203. However, it is plausible that end-users would interpret the range to represent the credible interval that the analyst assigned to the specific event—namely, “the Blanks will attack our country in the next week.” Thus, the end-user might be inclined to misinterpret the meaning of the numeric range presented.

To make matters potentially worse, the analyst in our hypothetical example might have had in mind a different credible interval. Indeed, the analyst’s credible interval might span more than one of the stipulated ranges. For instance, if pressed for a credible interval, the analyst might have assigned a range of 75%-85%. In reference to the ICD 203 standard, this range spans the stipulated ranges associated with the terms *likely* and *very likely*. Some analysts with such a range in mind might use the more extreme term (i.e., *very likely*), but perhaps more often than not they would choose the less extreme term. At least, this is suggested by work showing substantial underconfidence in intelligence analysts’ probabilistic forecasts [31,32].

An alternative approach for intelligence organizations and other organizations tasked with providing expert probabilistic judgments to end-users would be to report only the numeric probability ranges and forego the use of linguistic probabilities entirely (e.g., [11–13]). If this approach were adopted, the ranges could unambiguously refer to the analyst’s credible interval. In a recent experiment involving 1202 crowdsourced online participants, [33] examined mean agreement with another intelligence standard used by NATO in intelligence doctrine. Participants were presented with a hypothetical intelligence assessment that either used only verbal probabilities (akin to Wintle *et al*.’s control condition), a combination of linguistic probabilities and numeric ranges (akin to Wintle *et al*.’s brackets condition) or only numeric probability ranges. The authors found that agreement tested with multiple measures was significantly better in the brackets and ranges-only conditions than in the verbal-only condition. Those findings reinforce recommendations to use numeric probabilities as a basis for communicating probability judgments in intelligence and other domains of expert judgment.
